# Neurobehavioral assessments and biomarkers in school children who are highly exposed to air pollution

**DOI:** 10.3389/fpubh.2026.1970714

**Published:** 2026-10-14

**Authors:** Stephanie M. Holm, Nina Holland, John R. Balmes, Tim Tyner, Weihong Guo, Anabelle Garza, Debra Manzo Garcia, Briseida Vasquez-Vasquez, Sierra Loomis, Elizabeth M. Noth

**Affiliations:** 1 Stephanie Holm Consulting, Vancouver, BC, Canada; 2 Division of Environmental Health Sciences, School of Public Health, University of California, Berkeley, Berkeley, CA, United States; 3 Division of Occupational, Environmental and Climate Medicine, Department of Medicine, University of California, San Francisco, San Francisco, CA, United States; 4 Central California Asthma Collaborative, Fresno, CA, United States; 5 Division of Epidemiology and Biostatistics, School of Public Health, University of California, Berkeley, Berkeley, CA, United States

**Keywords:** adolescent, air pollution, brain-derived neurotrophic factor, child, cognition, neuropsychological tests, polycyclic aromatic hydrocarbons

## Abstract

**Background:**

Children are uniquely vulnerable to ambient air pollution. Yet, data remain limited relating standardized academic metrics with neurobehavioral outcomes measured by more in-depth testing, and how neurobehavioral outcomes are associated with non-criteria pollutants—such as polycyclic aromatic hydrocarbons (PAHs) and elemental carbon (EC).

**Methods:**

Utilizing data from the Children’s Health and Air Pollution Study (CHAPS) in Fresno, California, we evaluated neurobehavioral outcomes in a cohort of highly exposed children (*n* = 193, ages 7–18). Prenatal and year of life one exposures to PM_2.5_, PM_10_, O_3_, NO_2_, NO_x_, PAH_456_ and elemental carbon (EC) were estimated daily. Outcome measures included the NIH Toolbox Cognition Battery, the Child Behavior Checklist (CBCL), standardized test scores, and serum brain-derived neurotrophic factor (BDNF) and urinary 8-hydroxy-2-deoxyguanosine (8-OHdG) biomarkers. Correlations between the various outcomes were assessed, and linear and distributed lag models (DLM) associated outcomes with pollutant exposures.

**Results:**

Third-grade test scores demonstrated robust correlations with the NIH Toolbox total and crystallized cognition composites (*r* ~ 0.55–0.60) and moderate correlation with BDNF (*r* = 0.35). Linear models revealed that first-trimester exposure to traffic-related air pollutants was associated with elevated aggressive and rule-breaking behavior. First-year of life exposure across all pollutants was associated with depressed BDNF levels with a susceptibility window around 5 to 6 months of life.

**Conclusion:**

Early-life exposure to ambient air pollution is associated with persistent differences in neurocognitive and behavioral outcomes measured into adolescence. The strong concordance between school-administered standardized test scores and resource-intensive neurobehavioral tools indicates that academic datasets might provide proxy endpoints for assessing environmental neurotoxicity.

## 1 Background/context

Children are especially vulnerable to ambient environmental hazards because differences in their activity levels and physiology mean that they are exposed to a higher effective dose of air pollution (1). Because childhood is characterized by rapid change and development, oxidative insults during childhood can disrupt growth patterns and may have life-long consequences (1–4). There are many known adverse health effects of air pollutants, including mounting evidence for neuro-developmental effects in childhood (1).

In addition to air pollution exposure throughout childhood and adolescence, exposure during the prenatal period (when there is rapid growth of brain structures) may have effects that extend into adolescence. Functional connectivity of the human fetal brain increases substantially in the second half of pregnancy (5, 6). Higher prenatal fine particulate matter (PM_2.5_) exposure has been associated with lower cognitive functioning in early childhood (ages 1–6) (7–10), as has prenatal exposure to larger diameter PM (ages 2–6) (11, 12), to polycyclic aromatic hydrocarbons (PAHs; aged 5) (13), to nitrogen dioxide (NO_2_; age 7) (14), and to roadway proximity (age 7–8) (14, 15). Though a large re-analysis of multiple European birth cohorts did not find any relationship between *in-utero* PM_2.5_ and cognitive function (16), exposure misclassification may have obscured a relationship (air pollutant data used were collected many years after the presumed exposure). An association was recently demonstrated between prenatal PM_2.5_ exposure and intelligence quotient (IQ) in 10-11-year-olds, the oldest cohort in which prenatal exposure–based on air pollutant data collected at the time of the presumed exposure–has been associated with cognition (17).

Notably, there are very few studies that have examined the cognitive effects of non-criteria pollutants—pollutants not monitored and regulated by the Environmental Protection Agency (EPA), such as PAHs and black/elemental carbon (BC/EC)—especially for exposure in the prenatal period. PAHs are carcinogenic (18–20), toxic air contaminants (21) that are co-emitted with regulated gaseous and particulate combustion products (PM, NO_x_, CO) from common sources, such as motor vehicles and biomass burning (22–25). Human exposure to PAHs is associated with adverse health outcomes, but the extent and trends in environmental concentrations are poorly understood, as no state or national agency requires routine monitoring of PAHs (26–29). PAHs are also classified as Persistent Organic Pollutants (30), meaning that they remain present in the environment for long periods, which makes it even more crucial that we understand what the effects of our PAHs will be on this and future generations. Yet, all the known relationships between prenatal PAH exposure and childhood neurodevelopment come from only two cohorts, both of which used a single snapshot of PAH exposure during pregnancy [either a 48-h personal monitor (31–33) or umbilical cord DNA adducts (13)] to estimate exposure for the full prenatal period. PAH exposures can vary by more than an order of magnitude throughout pregnancy (27), so single measurements are unlikely to capture the full variation in exposure. Furthermore, researchers generally report only particle-phase dominant PAH concentrations (31–33). But excluding vapor-phase PAHs underestimates total PAH exposure by up to 80% (34) and also emphasizes the exposure to the larger, less volatile PAHs. Existing research on BC–which is often used as a tracer for ultrafine, diesel exhaust particles– and cognition is relatively mixed. Some studies have demonstrated decreased cognition in childhood with either total lifetime or recent BC exposures (35–37), yet a few studies have found no relationship (15, 38, 39), potentially because of inadequate exposure estimates during critical windows of development or crude spatial resolutions for their exposure (39).

Cognition and neurodevelopmental growth are complicated constructs that can be assessed in a variety of ways, including those that assess general cognitive abilities as well as ones that assess specific cognitive domains such as attention, language, and memory (40). A recent systematic review (2022) of traffic-related air pollutant (TRAP) exposure (41), either prenatally or postnatally, and neurodevelopmental outcomes in children focused on detailed metrics such as IQ, executive functioning, learning and memory domains, and motor skills. Those authors concluded that there was a negative effect of PAH, PM_2.5_ and NO_2_ on children’s cognitive functioning, particularly attention and executive functioning, as well as overall intelligence. Yet limited numbers of children complete such detailed cognitive assessments, because they are time and resource intensive. Though not designed to assess broad cognitive growth, standardized testing is completed by most American children through their schooling. Multiple recent studies have in fact demonstrated relationships between air pollution and school (42) or district (43, 44) level academic outcomes. A 2021 systematic review of TRAP exposure and academic outcomes (including standardized testing results) found evidence of a negative association between TRAP and test scores, though the authors noted that the overall quality of the evidence was low (45). Thus, there is a need for work that combines both standardized testing outcomes and more detailed neurobehavioral assessments in children highly exposed to air pollution to tie together these disparate portions of the literature, and to help us understand whether standardized testing—the more widely available endpoint—could be used as a potential proxy for other cognitive outcomes.

Assessment of neurologically relevant biomarkers can also inform the biological processes that may underpin air pollutant-cognitive function relationships. Commonly measured biomarkers of effect include 8-hydroxy-2-deoxyguanosine (8-OHdG), a marker of oxidative stress (46, 47) and Brain-Derived Neurotrophic Factor (BDNF) a biomarker of neurogenesis. 8-OHdG is produced by hydroxylation of guanine–a type of DNA oxidative damage– and has been associated with a variety of health outcomes including psychiatric disorders (48, 49). 8-OHdG (50–54) has been specifically associated with second-hand tobacco smoke, as well as to exposure to pollutants like ozone, PM and PAH in children and adults, including with seasonal variability (50, 51, 55). BDNF is a growth factor that regulates neuron plasticity and differentiation and may be particularly important for cognition and learning (56, 57). BDNF has been considered as an informative biomarker for cognition and neurodegeneration in adults and has been used in research of neurotoxicity from environmental toxins such as pesticides and lead exposure (58–60), but not yet with air pollution.

Because adverse neurobehavioral effects are likely to be most severe in highly exposed children, there is a particular need to assess these relationships among populations with high air pollutant exposures. Compared to the rest of the U. S., the Fresno metropolitan area (the largest city in California’s San Joaquin Valley) has both extraordinarily high ozone and particle pollution and fewer students meeting grade-level standardized testing standards. These disparities are particularly stark when comparing math and science results; in 2018–2019 (the last year with complete testing data prior to the COVID-19 pandemic), 18% of Fresno students met or exceeded state math standards, and only 16% of students met or exceeded science standards in Fresno (61). Given that Fresno has some of the most socioeconomically vulnerable communities in California (62) and that childhood cognitive growth is known to be associated with future earnings (63), any contribution of air pollutant-associated changes in neurodevelopment and associated cognition would be expected to have substantial lifetime impacts for members of this community.

Because our research team has worked extensively in the Fresno area, we are able to estimate daily exposures to PM_2.5_, PM_10_, O_3_, NO_2_, NO_x_, 4-, 5- and 6- ringed Polyaromatic Hydrocarbons (PAH_456_), and BC—many of which are traffic related—including during the critical prenatal and early-life exposure windows, in this particularly exposed population. This analysis combines those robust exposure data with the following outcomes measured in childhood or adolescence: detailed neurobehavioral assessments, biomarkers and standardized testing results. We hypothesized that higher air pollutant exposure would be associated with worse neurobehavioral outcomes and that the various ways of measuring neurocognition could be correlated with each other.

## 2 Methods

### 2.1 Study population and recruitment

The Children’s Health and Air Pollution Study (CHAPS) includes two pediatric environmental health cohorts that were assembled in the San Joaquin Valley of California and have been followed for multiple years. The CHAPS1 cohort children were recruited from a local medical center where the parent was receiving prenatal care at 18–25 weeks in utero between 2015–2017 (original enrollment n = 225), and were seen at roughly 12 months, 24 months, 7.5 years and 8.5 years old. The CHAPS2 cohort children (original enrollment n = 299) were recruited at ages six-eight from 2015 to 2016 from first and second grade classes in the Fresno area and seen at roughly 7 years, 9 years, 15 years and 16 years. No residential stability requirement was included during recruitment, to avoid excluding lower income families. Historically about 70% of CHAPS cohorts were retained between contact periods, though retention was lower during the COVID-19 pandemic. Notably, the loss reflects an inability to reach prior participants, consistent with this being a lower-income, highly mobile cohort, rather than participation refusal (e.g., at the CHAPS2 13–16-year visits, 41% of participants returned after the COVID pandemic, 53% of participants were unreachable, and only 6% chose to withdraw when reached). As reported in a prior paper, those lost to follow up were more likely to be below the poverty line, and to self-identify as Black, but had generally similar air pollutant exposure distributions when compared to those who remained in the study (64).

This paper reports on neurobehavioral assessments that were added to the study as part of the Children’s Health and Air Pollution Study-Standardized Assessments and Cognition in Kids (CHAPS-STACK). 193 participants completed a CHAPS-STACK visit between 2024 and 2025 at the field office site at Central California Asthma Collaborative in Fresno, CA (at one of the participants’ last two CHAPS visits, see Figure 1). The CHAPS project is approved by the University of California Berkeley Institutional Review Board. Written informed consent and permission was received from each accompanying parent, and written assent was received from all children.

**Figure 1:**
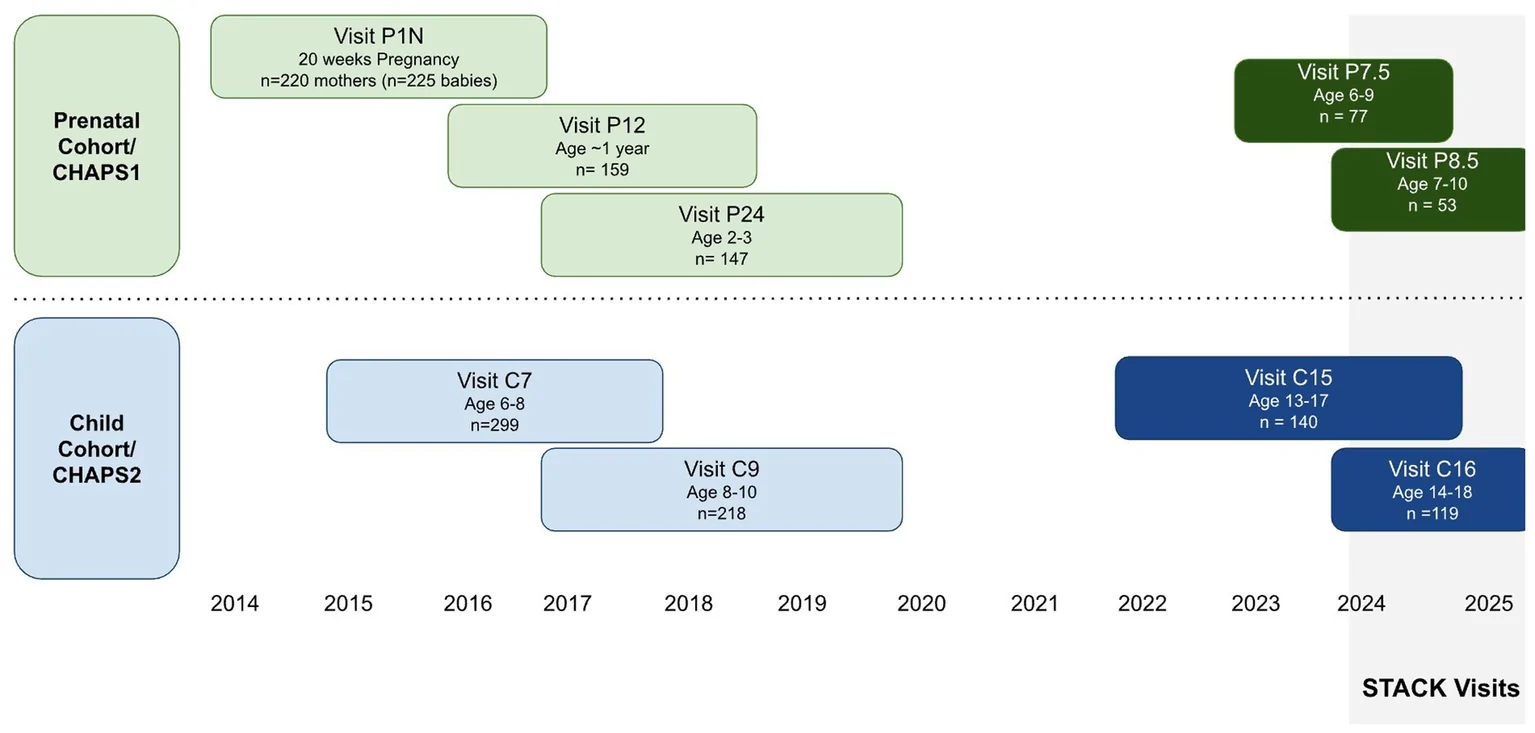
Children’s health and air pollution study diagram, both overall and neurodevelopmental (“STACK”) visits.

### 2.2 Air pollution modeling

A combination of field monitoring and predictive modeling was used to estimate exposures for each of the participants for the entirety of their gestational period and their first year of life (births occurred between 2006 and 2016). PM_2.5_, PM_10_, and ozone (O_3_) data were interpolated from the EPA’s Air Quality System (AQS) ambient data using inverse-distance-squared weighting. The model used up to four sites, with the maximum allowed radius from each address being 50 km. Linear regression air pollution exposure modeling was used to develop a daily spatial–temporal model for NO_2_, EC, and the sum of 4-, 5-, and 6-ring PAH compounds (PAH456) using field monitoring data from Fresno and Clovis, as described in prior papers from our team (27, 65). Exposure regression model covariates included aggregated particle phase-PAH and NO_2_ measured continuously at fixed sites, relative humidity, temperature, wind speed, atmospheric stability, distance to nearest freeway, Caline4 dispersion model estimates of concentrations from local traffic, distance to nearest rail lines, and amount of rail yard, urban, and open space land use within 1 km radius circular buffers. Linear regression models with sampling location and sampling date as random effects were used to simultaneously model the temporal and spatial components. The inclusion criteria for covariates were their statistical significance and percentage of covariance explained (i.e., improvement in explanatory power). Further information about exposure models can be found in a previous paper (65).

### 2.3 Exposure assignment

During their visits to the study center for health outcome measurements, parents/guardians were asked to report the street address, city, and state of all residences where the pregnant parent resided during the gestational period, and the dates they were at each. Each address was geocoded using ArcGIS (ESRI, Redlands, CA) to create a residential history throughout the pregnancy.

For the CHAPS1 cohort, pregnancy addresses were ascertained during pregnancy or in the first year of life, with addresses reviewed and any gaps in address history filled at later visits. For the CHAPS2 cohort pregnancy addresses were reported by the parent retrospectively at child or adolescent visits. Daily ambient (i.e., outdoor) residential pollutant concentrations for each day were estimated using the residential location (parental residence during pregnancy or child residence during the first year of life) on the day of interest and the corresponding modeled daily value of each pollutant for that location. Concentrations were aggregated for each participant for each trimester of the gestational period, and each month during the first year of life. Daily ambient concentrations were averaged to create an estimate across the whole pregnancy and across the entire first year of life.

### 2.4 Covariate measurements

Child-specific covariates used in this analysis were collected at the child’s two recent study visits. At both visits, the child’s parent or guardian was interviewed using an in-depth questionnaire offered in either English or Spanish. Older youth (>10 years) were also asked privately about their own potential smoking behavior.

Race was self-reported by participants, choosing between “White,” “Black or African American”, “Hispanic or Latino”, “Asian”, “Native American or Other Pacific Islander”, “American Indian or Alaskan Native”, “Mexican”, “Middle Eastern or Arab”, “Some other group”, and “I don’t understand this question”. Due to the low number of participants in other groups, these were further summarized as Hispanic, non-Hispanic white or non-Hispanic other, as has been done previously in this cohort (64, 65). At both visits, parents or guardians were asked about their household income, their home ownership status, their income needs, and their education. Reported household incomes were categorized as “above or below $30,000”. Parents or guardians were further interviewed about smoking in the child’s home and the cars in which the child frequently rode, and whether the child was exposed to smoking was categorized as “yes” or “no”.

Two covariates were used to represent the timing of the office visits. These included which season the visit occurred in and the age of the youth on the visit date (calculated from their parent-reported date of birth). Consistent with weather patterns in Fresno, visit season was described as either the hot season (April through October) or the cool season (November through March).

### 2.5 Outcome measurement

At recent CHAPS visits, participants had anthropometrics and blood pressure measured, biospecimen samples collected (urine, blood, hair, nails, saliva), and performed lung function testing (spirometry). At the CHAPS-STACK visit, the youth additionally completed several neurobehavioral assessments, which were used for these analyses.

The NIH Toolbox Cognition Battery, a validated and widely used set of measures that allow for continuity of cognitive assessment across a wide variety of ages (7–85 years) (66), was administered in the office via an iPad app. It consists of seven standard but adaptive computerized instruments, and study staff were trained in the use of this tool by a pediatrician and a neurodevelopmental psychometrist, per the guidance in the administration manual (67). Per the technical manual, the cognition battery composite scores have excellent empirical reliability with change sensitive scores in pediatric populations of 0.98–0.99, and a correlation coefficient of 0.64 when compared to full scale IQ testing (68). The participants completed 7 tasks (list sorting working memory, oral reading recognition, picture vocabulary, flanker inhibitory control and attention, dimensional change card sort, pattern comparison processing speed, and picture sequence memory). Collectively, these tasks assess executive function, attention, working memory, language, processing speed, and episodic memory. If the participant was able to complete the full set of tasks required for each composite score, the software calculated three composite cognition scores: fluid cognition, crystallized cognition, and total cognition. The fluid cognition component is intended to represent cognitive processes, whereas crystallized cognition is a measure of knowledge. A higher score on these composites indicates better cognitive functioning. In the validation sample for this battery of tests, fluid and crystallized cognition are moderately correlated with a correlation coefficient of 0.35; total cognition is highly correlated with both individual domains (total to fluid *R* = 0.86, total to crystallized *R* = 0.78).

For all participants, the parent also completed the multiple-choice portion of the Child Behavior Checklist (CBCL) (69), a widely used assessment of behavioral and emotional challenges. Children over age eleven also completed a companion survey, the Brief Problem Monitor (BPM). The software calculates 8 standardized syndrome scales from the CBCL based on a factor analysis derived from the validation sample (Anxious/Depressed, Depressed, Somatic Complaints, Stress Problems, Thought Problems, Attention Problems, Rule-breaking behavior, and Aggressive behavior) and 3 from the BPM (Internalizing Problems, Externalizing Problems, Total Problems). A higher score on these scales indicates more problems with the specific domain. In the normative sample for these tests, test–retest reliabilities for the CBC syndrome scales used range 0.82–0.9. As recommended in the manual for these forms, raw values were standardized within 6–11- and 12-18-year age groups, and these standardized scores were used for analysis.

Two biological markers relevant to brain growth and development were also assessed from the biospecimens collected at these visits: serum brain derived neurotrophic factor (BDNF) and urine 8-hydroxy-2′-deoxyguanosine (8-OHdG). Both BDNF and 8-OHdG were measured using Enzyme-Linked Immunosorbent Assay (ELISA) kits according to protocols from their respective manufacturers (ABCAM and Invitrogen-ThermoFisher Scientific) and as previously described (52, 70, 71). All samples were run in duplicate; QA/QC samples included method blanks, coded repeat samples, and internal controls. All ELISA plates were read on the SpectraMax plate reader at 450 nm (Molecular Devices, San Jose, CA) and analyzed using SoftMax Pro 7.1.2. To achieve reliable and accurate results, repeated runs were performed for samples with higher coefficients of variation (CV) or for sample concentrations outside of the calibration range by changing dilution factors. All calibration curves had an R^2^ > 0.99. For BDNF, the limit of detection (LOD) was 0.0024 ng/mL, and the mean CV was 2.9%. For 8-OHdG, the LOD was 0.94 ng/mL, and the mean CV was 7.1%.

In 2015, California began using an updated form of statewide standardized testing for English Language Arts (ELA) and math called the Smarter Balanced Assessments (SBAC) (61). The SBAC tests are administered in 3rd - 8th grade and in 11th grade. Testing is done in the spring semester of every school year. For the state of California, SBAC scores for 3rd - 8th graders range from 2,114–2,769 for ELA and 2,189–2,802 for math with higher scores indicating better performance. For any participants who previously completed California standardized tests, CCAC staff worked with parents to obtain prior testing results, most commonly by helping them navigate their online portal for download or copying from the parent’s own records. SBAC scores were available from third grade testing for 104 participants (68% of those far enough in school to have completed any standardized testing), and within the current or prior 2 school years for 52 participants (51% of those who were in grades that would be expected to have had testing results). Scores at the statewide level fluctuate slightly by year and grade based on the test characteristics and statewide period effects (such as COVID-19) each year. To adjust for these differences, we standardized the test scores in our sample to z-scores using the statewide averages and standard deviations for a given grade and year.

### 2.6 Statistical analysis

R version 4.5.3 (“Reassured Reassurer”) (72) was used for modeling, using the packages corrplot (73), dlnm (74), kableExtra (75), MASS (76), mice (77), openxlsx (78), tableone (79), and the tidyverse (80). We selected covariates *a priori* based on domain knowledge with the aid of a directed acyclic graph. Covariates selected included potential confounders related to both the exposure and the outcome (season of the study visit, parent identified race/ethnicity, household income, smoking in the home or car), as well as variables thought to be highly correlated with the outcome only (age at the study visit and child sex) (81).

The main analyses were done using linear regressions. The standard assumptions of linear models (linearity of the relationship, normally distributed residuals, and non-heteroskedasticity) were assessed with fitted v. residual and Q-Q plots. For the main analysis, 630 separate linear models were run: one for each of the 18 outcomes (3 from the NIH Toolbox, 8 from CBCL, 3 from BPM, 2 biomarkers, 2 SBAC) with each of the seven air pollutants (NO_2_, NO_x_, EC, PAH_456_, PM_2.5_, PM_10,_ O_3_), and each of 5 time points (trimester 1, trimester 2, trimester 3, the whole pregnancy, the first year of life).

The secondary analyses were done using distributed lag models (DLM), which allowed us to include monthly averages of exposure during the complete first year of life in a single analysis, thereby controlling for exposure at all other times within the year (74, 82, 83). Models were unable to converge if the relationship was allowed to be flexible in both directions. Thus, we assumed that the relationship between each pollutant and neurodevelopmental outcome was linear across our range of pollutant concentrations, but we allowed the shape of the time-lag dimension to vary flexibly. We examined multiple options for smoothing functions (including allowing non-linearity in the exposure dimension) and examined the Akaike Information Criteria (AIC) (83). The model specification with the best model fit (lowest AIC) used b-splines for the smoothing functions.

We did not calculate or report *p*-values and thus did not adjust for multiple comparisons in our models. Instead, we interpret the results of pollutant-specific models in the context of larger patterns.

Because associations between prenatal exposure and standardized testing scores showed effects that were quite different from the relationships with any of the other outcomes, a *post-hoc* sensitivity analysis was run incorporating the kindergarten cutoff date. Because of seasonality in pollution, the fact that pregnancies are less than a year, and that there are strong effects on educational attainment related to age at school entry, it would be possible for seasonal effects to confound the pollutant-outcome relationship for school-based outcomes. Children born between March 1 and the kindergarten entry date (that date ranged Sept1- Dec 2 depending on the child’s year of birth) were considered ‘young’ at kindergarten start and those born between December 3 and February 29 were considered ‘old’ at kindergarten start.

### 2.7 Missing data

To minimize bias resulting from missing data, we used multiple imputations to generate missing exposures and covariates. Imputation was done with version 3.19.0 of the “mice” package in R. Of the 195 participants, 7 were known to live out of the Fresno area during the exposure periods. Given that these participants would thus have had exposures that were not well represented by those in our dataset, exposure values could not be imputed for these. Of the remaining 188 participants, one or more summary measures (trimester averages, pregnancy average or first year of life average) had to be imputed for NO_2_, NO_x,_ and PAH concentrations for 24% of participants, EC for 48% of participants, PM_2.5_ for 19% of participants, PM_10_ for 22%, and O_3_ for 19% of participants. Across all participants, 4,105 out of 15,708 exposure-months (26%) were imputed. Most commonly data were missing for the first or last months of year one of life.

## 3 Results

In total, 193 youth (mean age 14 years, range 7–18; Table 1) completed a CHAPS: STACK visit. 48% of the cohort was male and 85% was Hispanic. This was a low-income population; only 58% of households had an annual income above $30,000, and half of families received government assistance. 12% of the youth were exposed to cigarette smoke in their home or family car. Though 20% of the youth themselves reported having tried smoking or vaping at some point, only 6% reported vaping in the last month, and 2% smoked in the last month.

**Table 1:** Demographic characteristics of the cohort.

Variables	Mean	SD	Count	Percent
n			193	100%
Study visit during the hot season			100	51.8%
Age	13.97	3.68		
Current grade at school				
Up to 2nd grade			22	12.6%
3rd to 10th grade			102	58.3%
11th to 12th grade			51	29.1%
Sex at birth				
Male			92	47.9%
Female			100	52.1%
Gender (parent reported)				
Boy			93	48.2%
Girl			99	51.3%
Transgender boy			1	0.5%
Race/ethnicity (parent reported)				
Hispanic			163	84.9%
Non-hispanic white			9	4.7%
Non-hispanic other race			20	10.4%
Annual household income			111	
More than $30,000 (%)				57.8%
Smoking				
Smoking in the home or car in the last month			24	12.4%
Kid reports ever smoking or vaping			38	20.1%
Kid smoked a cigarette in the last month			3	1.6%
Kid vaped in the last month			12	6.2%

These youth had a mean PM_2.5_ exposure of 16.3 μg/m3 ^3^ during their prenatal window and 16.6 μg/m3 ^3^ during their first year of life, above the current air quality standards, consistent with living in the Fresno area 7–18 years ago. The mean pollutant exposures for each trimester, the whole pregnancy, and the first year of life are presented in Table 2. During both the pregnancy and first year of life exposure windows, the NO_2_ and NO_x_ concentrations were very highly correlated (*r* = 0.95–0.95, Figure 2) and PM_2.5_ was highly correlated with NO_x_ and NO_2_ (*r* = 0.61–0.68). During the first year of life, EC was very highly correlated with O_3_ (*r* = 0.94), but they were only moderately correlated during pregnancy (*r* = 0.56). Comparing across the time periods, both ozone and EC during pregnancy were highly correlated with their own concentration during the first year of life(*r* = 0.92–0.93).

**Table 2:** Summary pollutant concentrations during the relevant time intervals (means and standard deviations).

Pollutants	Trimester 1	Trimester 2	Trimester 3	Whole pregnancy	First year of life
NO_2_ (ppb)	9.2 (3.8)	9.8 (5.2)	9.4 (4.3)	9.11(4.1)	9.4 (2.6)
NO_X_ (ppb)	21.7 (8.9)	22.3 (11.9)	22.0 (10.1)	21.5 (9.0)	21.8 (6.5)
EC (μg/m^3^)	1.2 (0.7)	1.2 (0.8)	1.2 (0.6)	1.2 (0.8)	1.2 (0.6)
PAH_456_ (ng/m^3^)	9.4 (3.3)	9.3 (4.3)	9.6 (4.1)	9.5 (4.1)	9.4 (1.7)
PM_2.5_ (μg/m^3^)	16.4 (5.8)	17.1 (8.0)	16.6 (6.6)	16.3 (6.7)	16.6 (3.2)
PM_10_ (μg/m^3^)	33.8 (6.6)	34.6 (8.1)	34.1 (8.1)	32.4 (7.6)	33.8 (4.2)
O_3_ (ppb)*	39.3 (16.5)	39.7 (17.7)	38.5 (19.1)	39.6 (19.9)	39.1 (12.9)

*ozone is the average of the 8-h daily maximum averages as reported in the regulatory networks, unlike the other pollutants that are averages of the entire daily concentrations.

**Figure 2:**
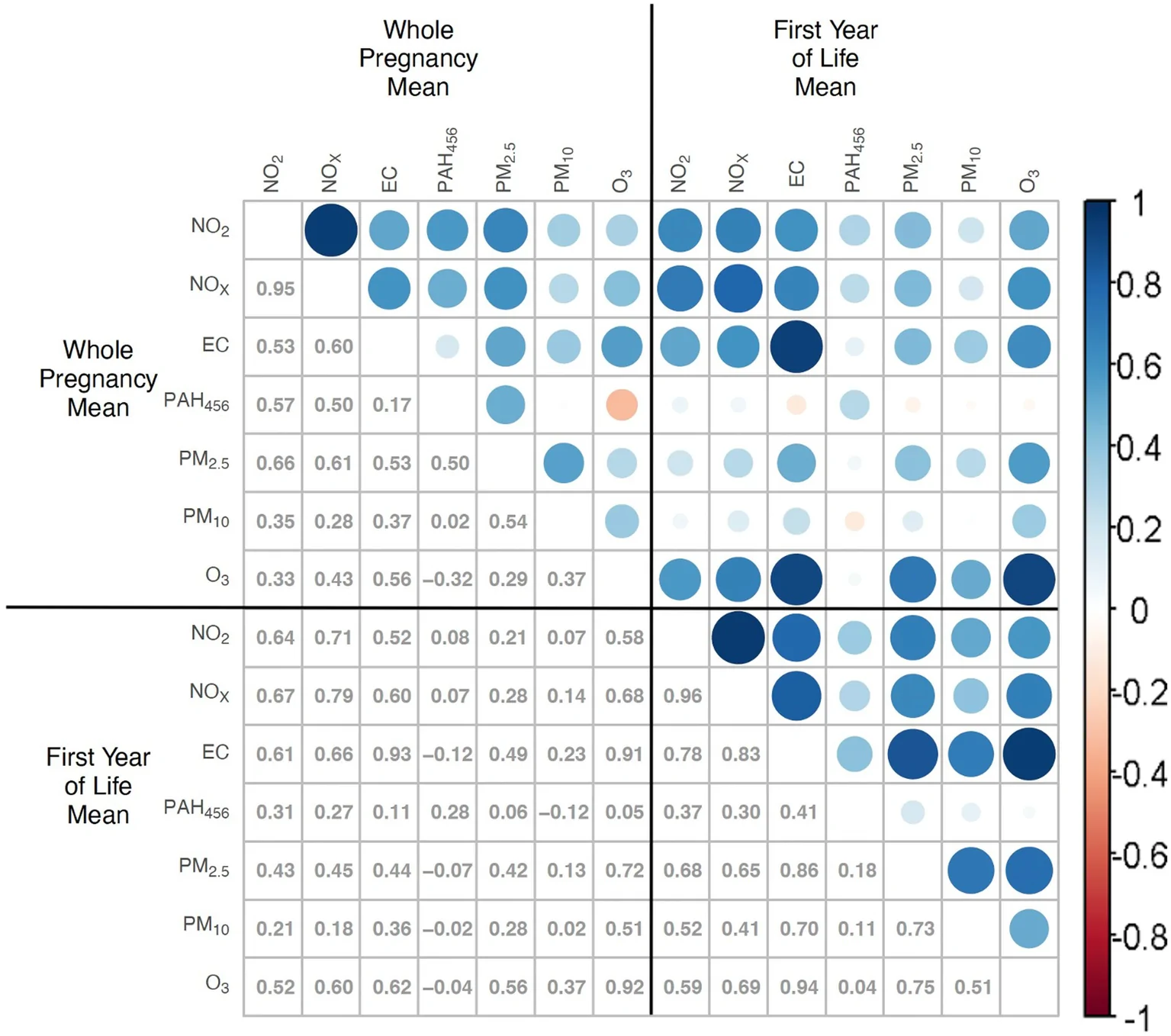
Correlations between different pollutants used in the models. Correlation coefficients are shown in the lower left, while those same correlations are represented with color and size in the upper right.

We were able to obtain nearly complete data for the tasks performed at our study visit. 191 youth (99%) had complete CBCL data and 134 youths completed the brief problem monitor (100% of the participants that were age-eligible). 187 completed sufficient tasks on the NIH Toolbox Cognition battery such that they had crystallized cognition composite scores (97%), and 174 (90%) additionally had fluid and total cognition scores. We obtained a sufficient blood sample for BDNF analysis in 164 participants (85%) and were able to measure 8-OHdG in urinary samples from all 193 participants. Of the 153 participants old enough to take standardized testing at school, 93 were able to locate SBAC scores from their 3rd grade school year (61%), and 52 (34%) had recent SBAC scores (within the last 2 years).

The various measures within each set of outcomes correlated well with each other (Figure 3). The total cognition composite score was strongly correlated with both the fluid and crystallized composites (*r* ~ 0.8), and the fluid and crystallized components were somewhat associated with each other (*r* = 0.34), all of which were very similar to the correlations reported in the validation samples for those tests. The different components within the child behavior checklist were highly correlated (most *r* > 0.5). The SBAC math and ELA scores taken at the same time were well correlated (*r* ~ 0.6).

**Figure 3:**
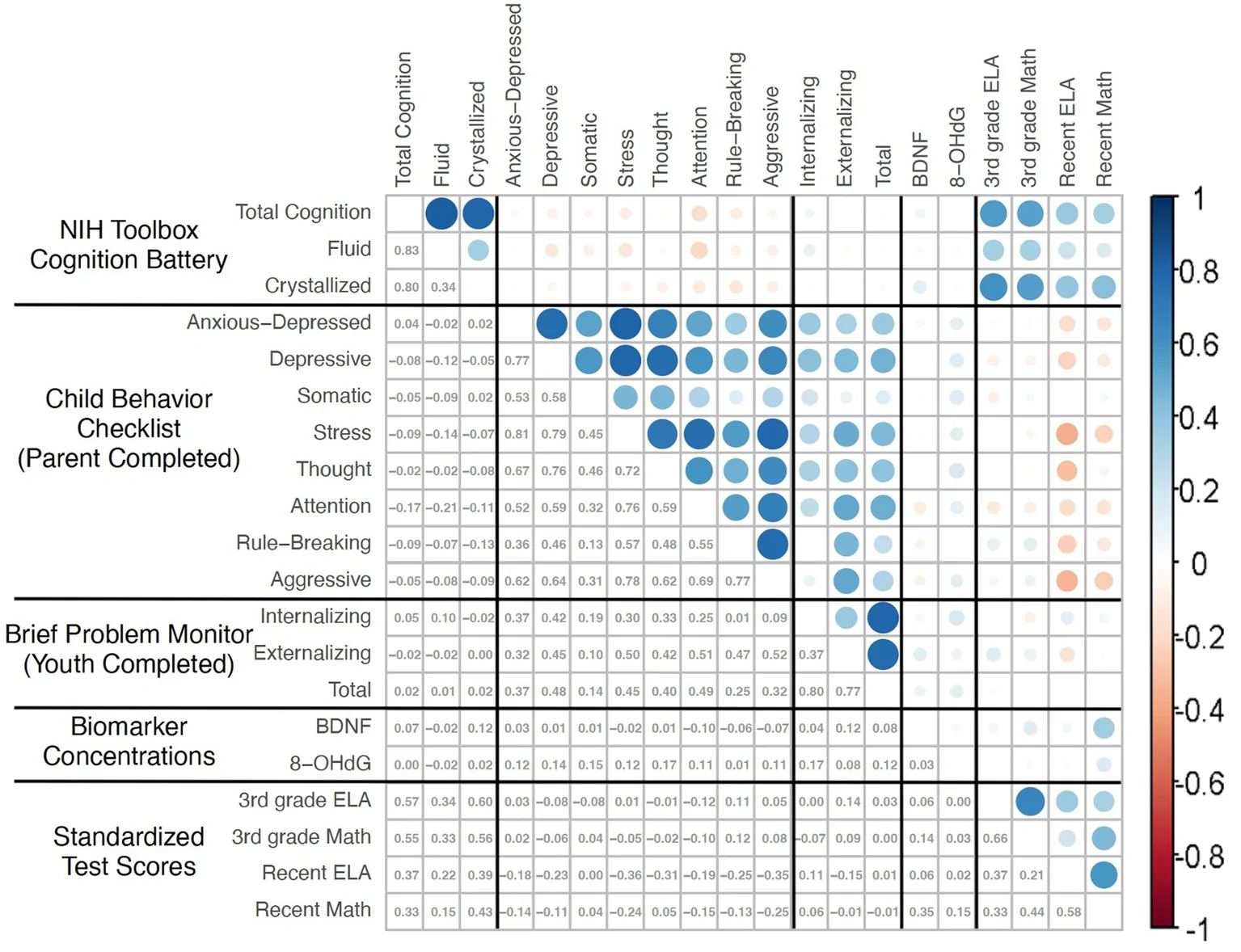
Correlations between different outcomes analyzed in the models. Correlation coefficients are shown in the lower left, while those same correlations are represented with color and size in the upper right.

When comparing across outcomes (Figure 3), the problems captured on the child behavior checklist (completed by the parent) had a moderate correlation with the brief problem monitor completed by the youth themselves; most correlation coefficients were in the 0.3–0.4 range. The child behavior checklist problems were weakly to moderately (and consistently) inversely associated with both the performance on the NIH toolbox and on the recent standardized testing results. The youth’s own report of their problems did not show a relationship with the NIH Toolbox nor standardized testing. 8-OHdG, the biomarker of oxidative stress, did not show a relationship with any of the other outcomes, including not showing a correlation with BDNF, the other biomarker. BDNF was moderately correlated with the youth’s performance on recent standardized math testing (*r* = 0.35). SBAC performance at third grade (on both subjects) was strongly correlated with both crystallized and total cognition as assessed by the NIH Toolbox (all *r* 0.55–0.6) and moderately correlated with fluid cognition (*r* ~ 0.35).

Exposure to multiple pollutants during the first trimester was associated with later rule-breaking behavior and aggressive behavior; the strongest associations were with PM_2.5_ and PAH_456_ (Figures 4, 5; Supplementary Table S1). 1 μg/m^3^ higher PM_2.5_ in the first trimester was associated with a 0.024 higher z score for aggressive behavior (95% CI 0.004, 0.044) and a 1 μg/m3 ^3^ higher PAH_456_ exposure during the first trimester was associated with a 0.043 higher z score for aggressive behavior (95% CI 0.008, 0.078). For rule-breaking behavior, 1 μg/m3 ^3^ higher first trimester PM_2.5_ was associated with a 0.022 higher z score for r behavior (95% CI 0.004, 0.039) and 1 μg/m3 ^3^ higher PAH_456_ with a 0.034 higher z score (95% CI 0.002, 0.065). There were no clear relationships with any of the other parent-reported scales, and no clear relationship between pollutant exposures during pregnancy or the first year of life and socio-behavioral problems reported by the youth (Figure 6; Supplementary Table S1).

**Figure 4:**
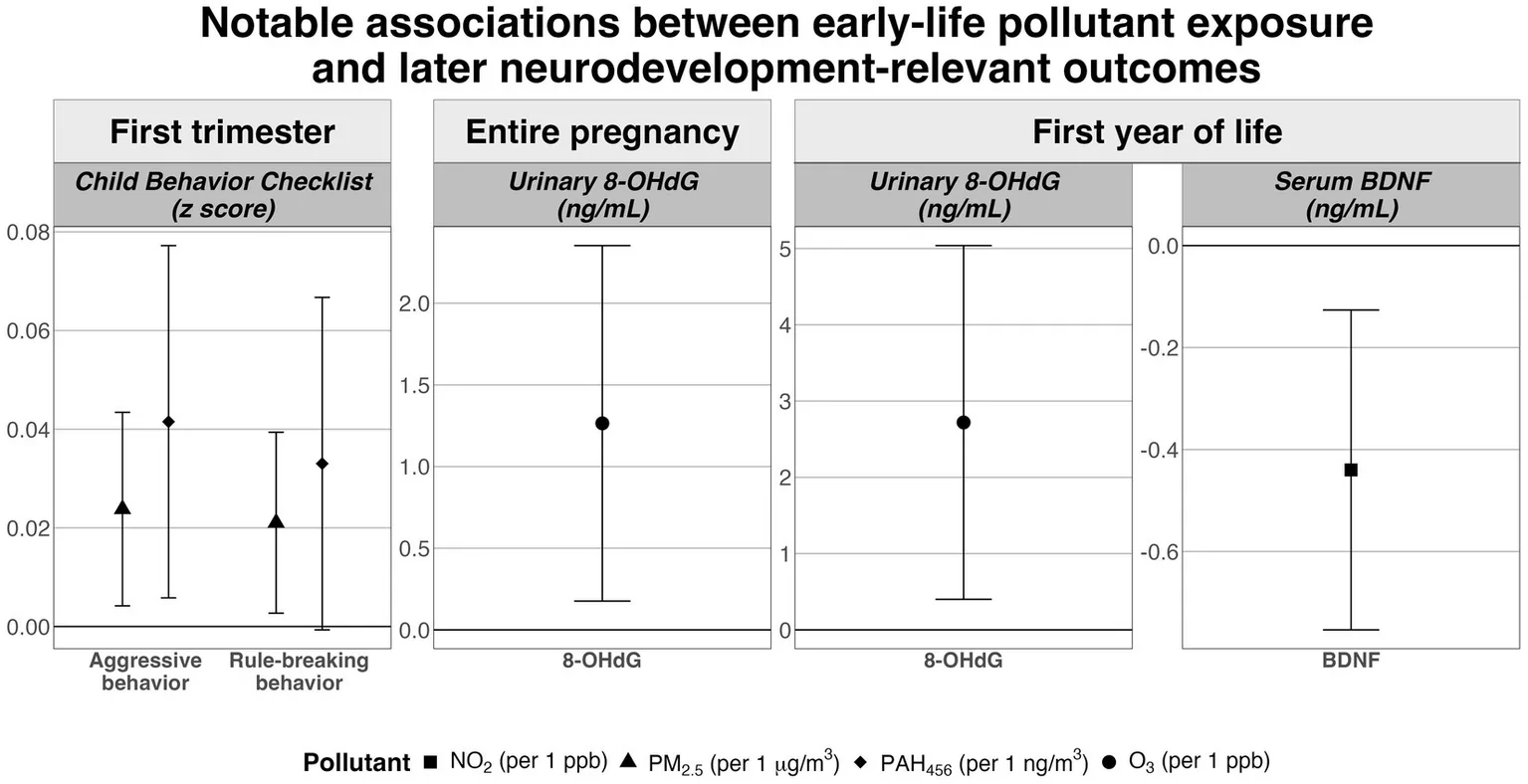
Notable associations between early-life pollutant exposures and neurodevelopment-relevant outcomes.

**Figure 5:**
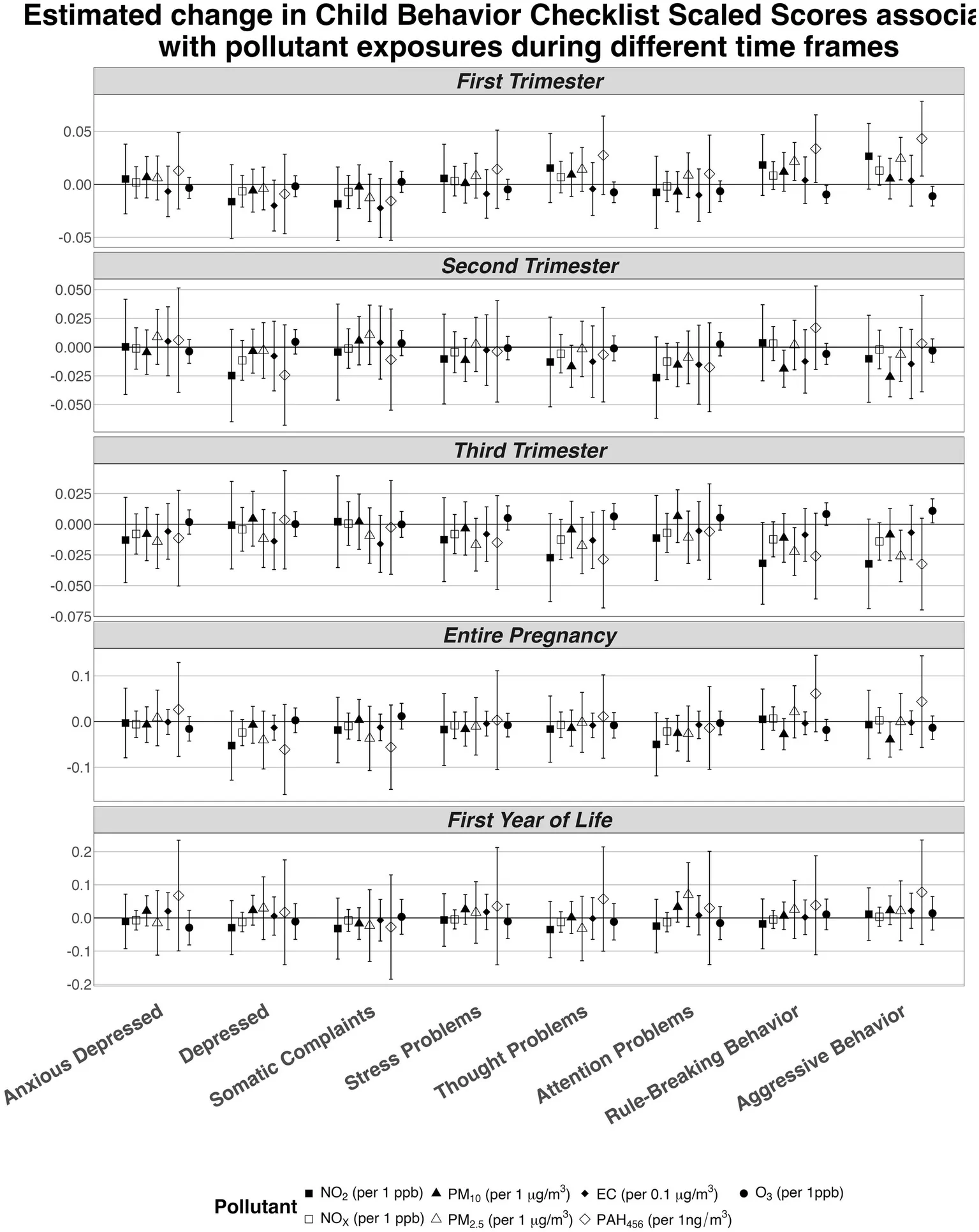
Estimated change in child behavior checklist scaled scores associated with pollutant exposures during different time windows.

**Figure 6:**
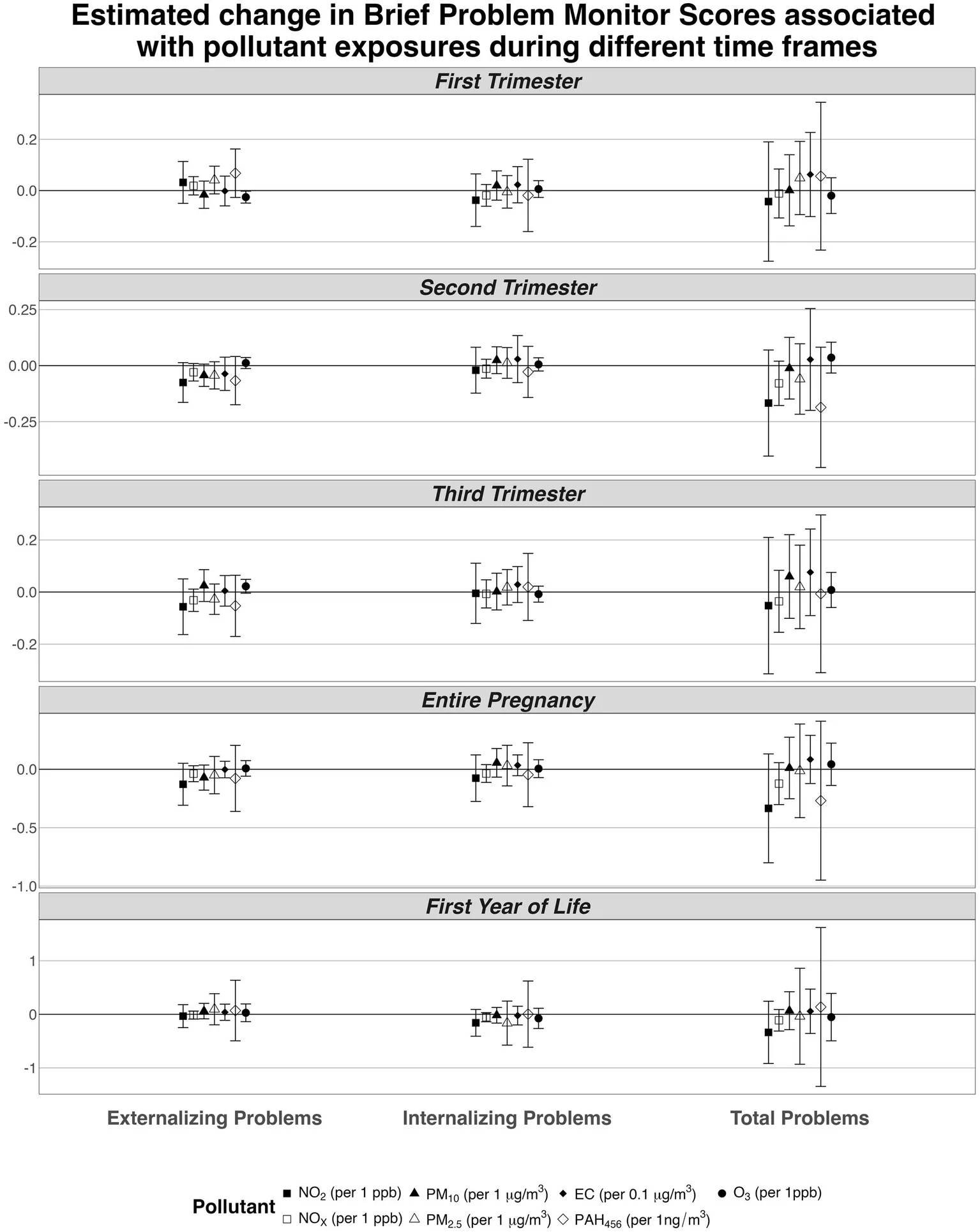
Estimated change in brief problem monitor scores associated with pollutant exposures during different time windows.

Using linear models to assess the relationship between NIH toolbox scores and mean pollutant exposures during pregnancy and the first year of life did not show any clear patterns with most time period-outcome pairings having a variety of pollutant effect estimates that clustered around the null (Figure 7, all linear model details are also in Supplementary Table S1).

**Figure 7:**
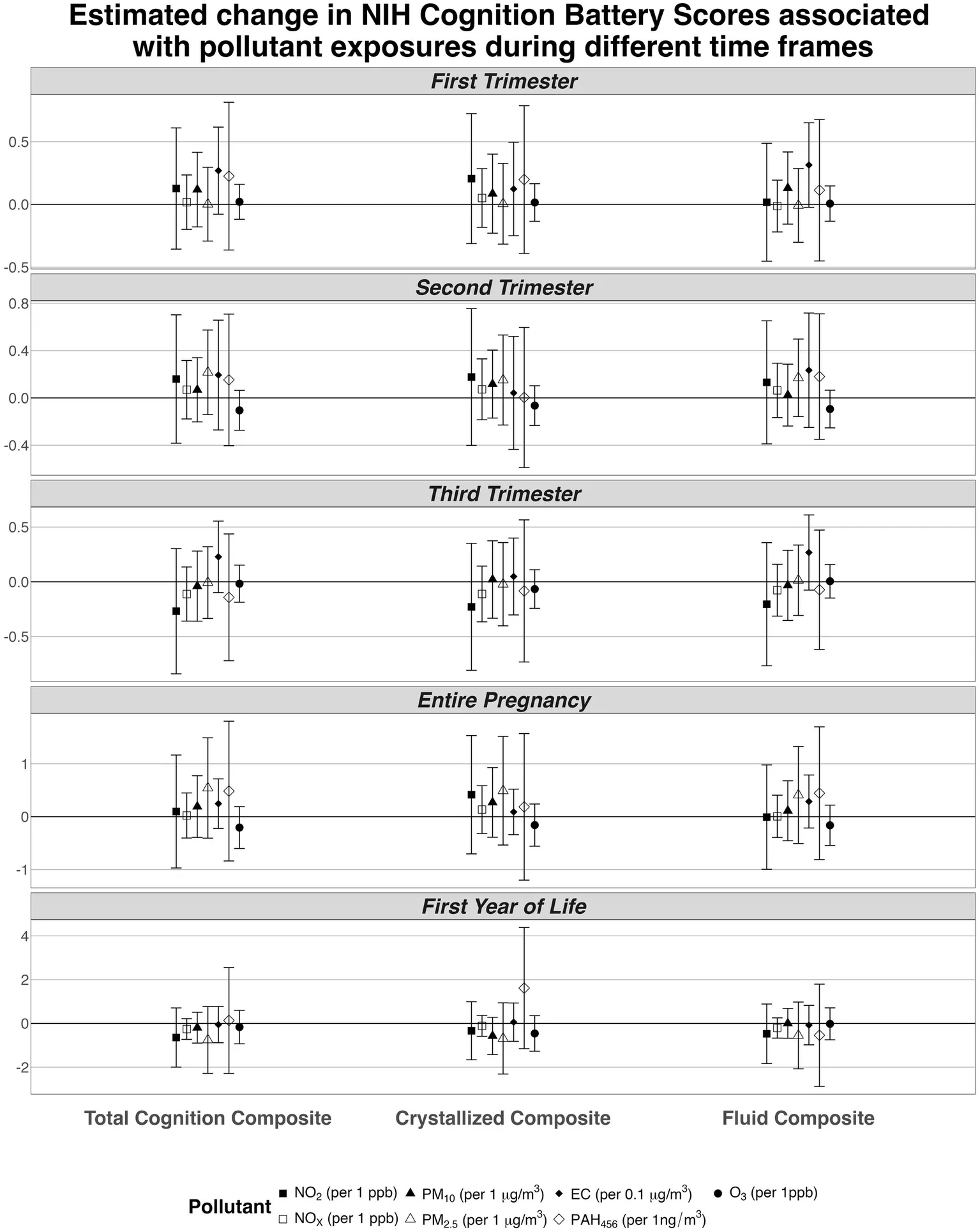
Estimated change in NIH toolbox cognition battery score associated with pollutant exposures during different time windows.

Exposure to all seven of the pollutants during the first year of life seemed to be associated with decreased levels of BDNF (Figures 4, 8; Supplementary Table S1). All seven relationships had negative estimated coefficients, many of which do not cross the null. For example, a 1 ppb increase in NO_2_ is associated with a −0.43 ng/mL decrease in serum BDNF concentration (95% CI −0.75, −0.10). Exposure during pregnancy did not show a clear pattern of relationships with serum BDNF concentrations. 8-OHdG concentrations were not as widely associated with pregnancy and early-life exposure (Figures 4, 9), but exposure specifically to 1 ppb higher average ozone during both the entire pregnancy and the first year of life is associated with increased 8-OHdG (1.01 ng/mL higher with pregnancy exposure, 95% CI −0.023, 2.035, 2.77 ng/mL higher with first year exposure, 95% CI 0.72, 4.82).

**Figure 8:**
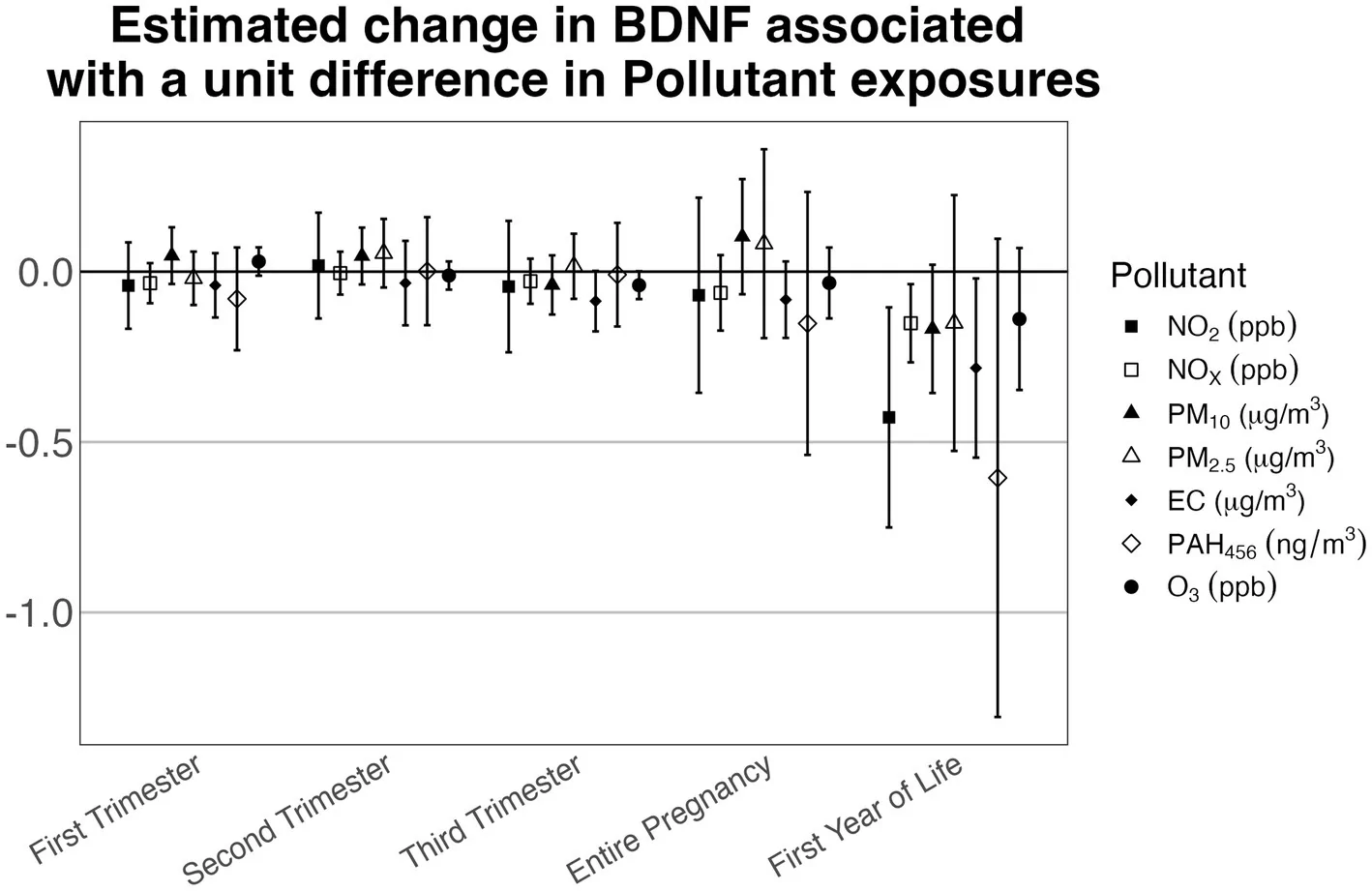
Estimated change in brain-derived neurotrophic factor serum concentrations associated with pollutant exposures during different time windows.

**Figure 9:**
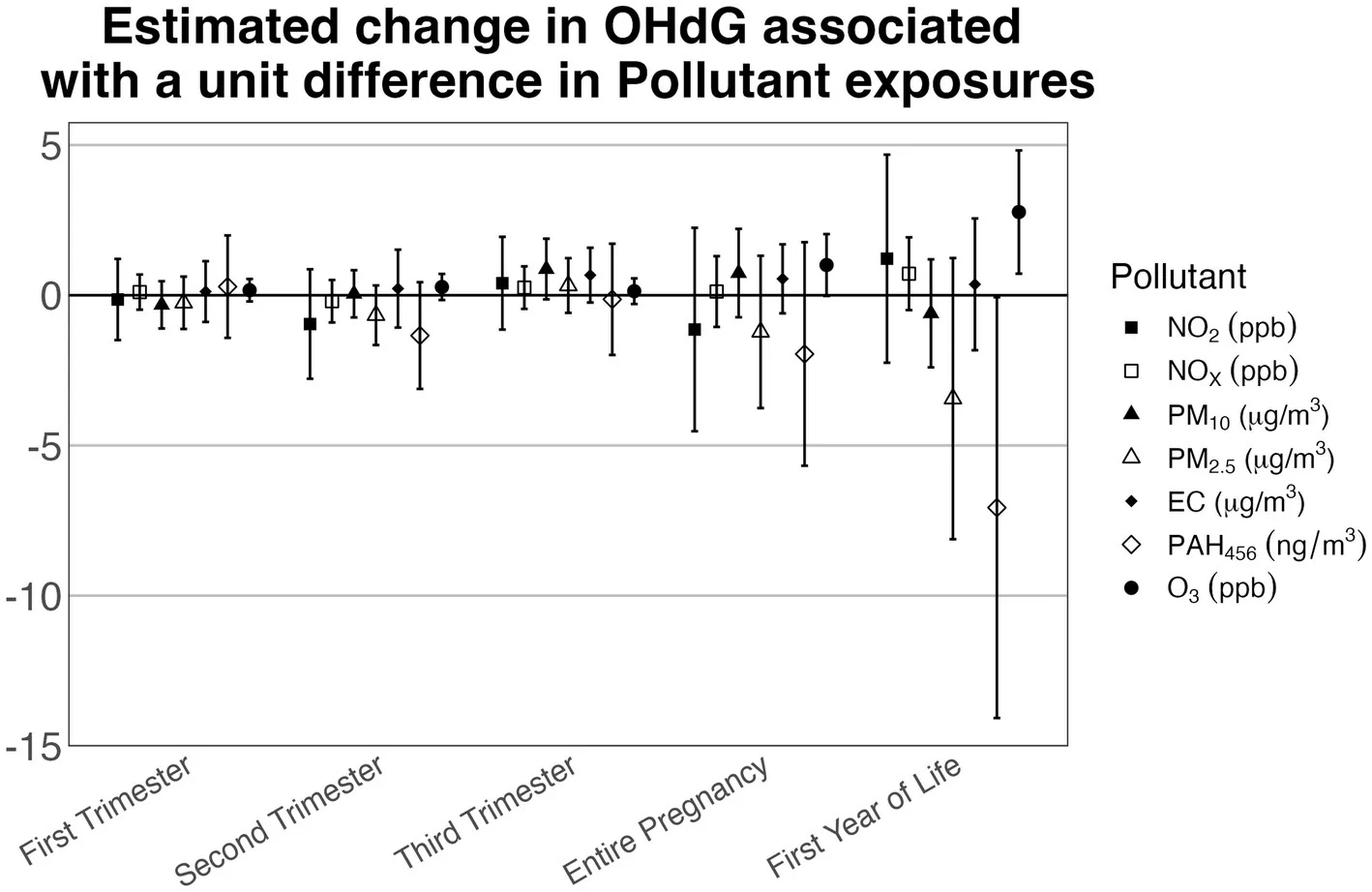
Estimated change in 8-hydroxy-2’deoxyguanosine urinary concentrations associated with pollutant exposures during different time windows.

Air pollutant exposures during the first year of life did not show clear relationships with standardized testing at grade three (Figure 10; Supplementary Table S1). However, higher pollutant exposure during pregnancy—and particularly higher pollution in the second trimester—was consistently associated with higher SBAC z-scores. Excluding ozone, exposure to any of the six other pollutants during first trimester, second trimester, and for the whole pregnancy (but not during third trimester) was associated with higher SBAC z-scores, with effect sizes ranging from 0.001 to 0.09. For example, 1 ppb higher second trimester NO_2_ was associated with a 0.040-z-score increase in SBAC ELA (95% CI 0.00, 0.080) and a 0.044-z-score increase in SBAC math score (95% CI 0.010, 0.077). Ozone showed the inverse relationship, with 1 ppb higher ozone in the second trimester associated with −0.010 lower z-score for SBAC ELA (95% CI −0.020, 0.001) and −0.008 lower SBAC math z-scores (95% CI −0.017, 0.000). However, a *post-hoc* sensitivity analysis that stratified children based on whether their birthdate was before or after the kindergarten entry date demonstrated that these relationships between air pollution exposure in pregnancy and third grade standardized test scores were very different within birthdate strata (Figure 11; Supplementary Table S2; Supplementary Figures S1, S2). Using the same example as above, among the 29 children who were “old” at the time they started kindergarten (meaning they had turned five sometime after the prior year’s kindergarten cutoff but before March 1) 1 ppb higher second trimester NO_2_ was associated with a −0.025 *lower* SBAC ELA z-score (95% CI −0.17, 0.12) and a −0.042 difference in SBAC math z-score (95% CI −0.149, 0.066). Whereas for the 50 children who were young at the time they started kindergarten (meaning they had turned five sometime between March 1 and the fall of that year) 1 ppb higher second trimester NO_2_ was associated with a 0.049 point higher SBAC ELA z-score(95% CI −0.007, 0.105) and a 0.059 higher SBAC math z-score (95% CI 0.018, 0.099). The ozone relationship also inverted, with a negative relationship with ozone remaining during the second trimester in those who started school young, but a positive correlation between ozone and scores among those who started school when they were older. With the stratification, the first trimester, third trimester, whole pregnancy and first year pollutant associations were generally null and very similar within strata. These sensitivity analyses suggested that the initial finding of a positive association between air pollution exposure during pregnancy and SBAC scores was confounded.

**Figure 10:**
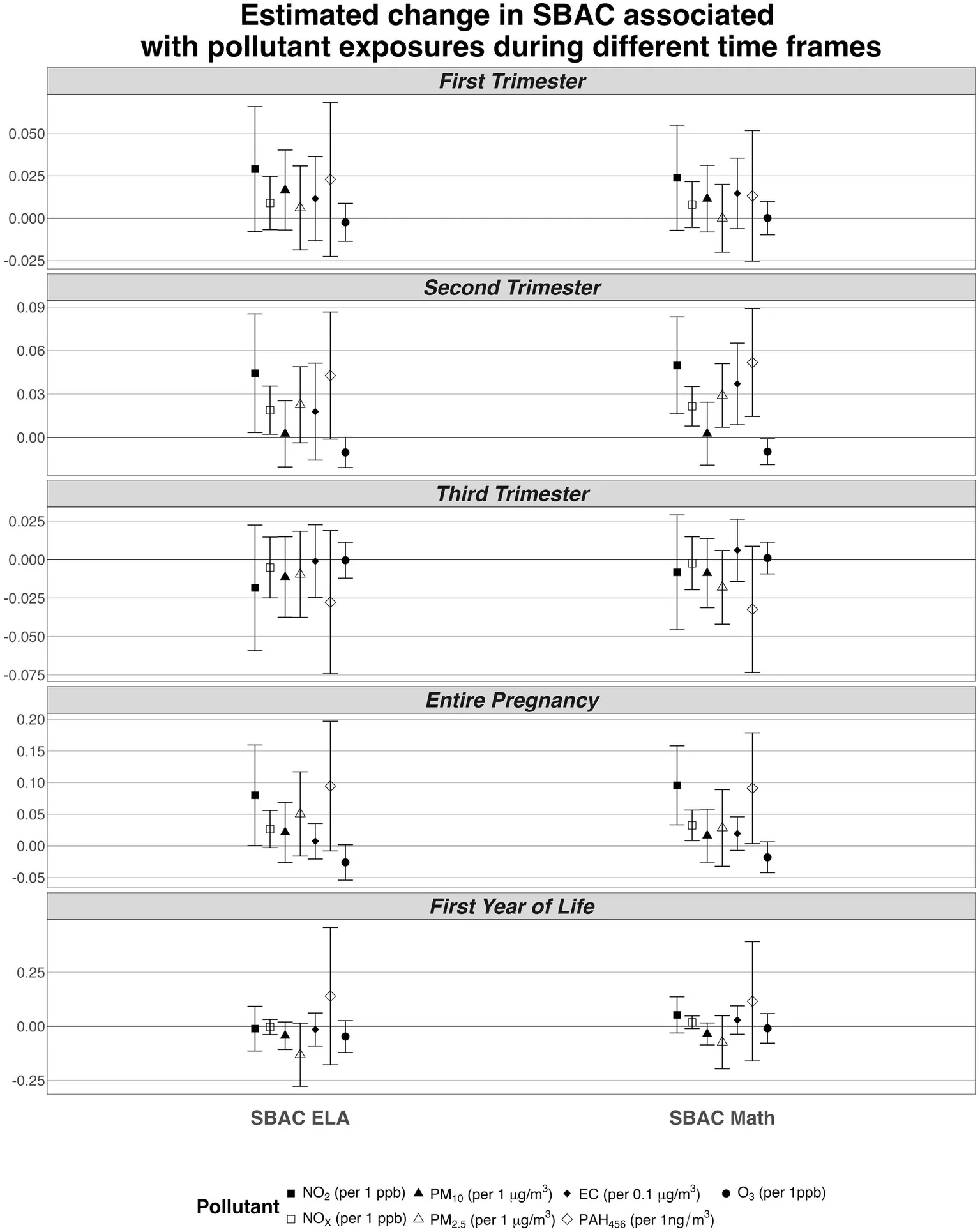
Estimated change in California standardized test scores (SBAC) associated with pollutant exposures during different time windows.

**Figure 11:**
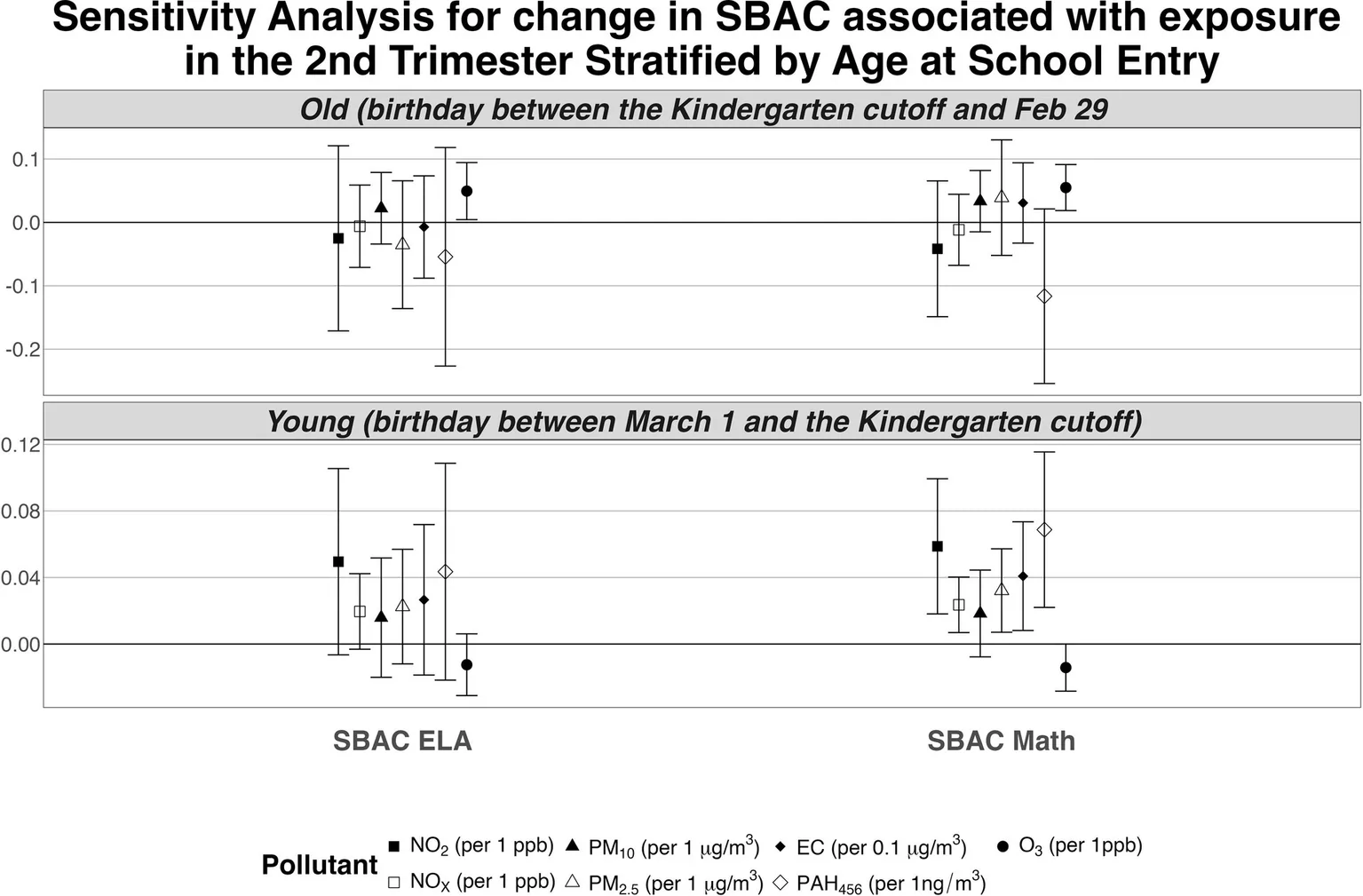
Sensitivity analysis for change in SBAC score associated with 2nd trimester pollutant exposure, stratified by age at school entry.

Distributed lag models were used to assess periods of exposure with larger estimated associations during the first year of life. For most outcomes, when controlling for exposures at all other months of the first year of life, no clear patterns were seen in which months were particularly important for exposure; point estimates hovered near the null and confidence bands widely spanned both sides of the null. However, for BDNF concentrations, exposure to NO_2_, NO_x_, particles, EC, and PAH_456_ all seemed to show a pattern of having the strongest negative relationships in the middle portion of the first year of life, with confidence intervals that did not cross the null for some time points for NO_x_ and NO_2_ and that were clearly asymmetric at the most affected time points for all of these pollutants. For example, a 1 ppb increase in NO_2_ during the child’s fifth month of life was associated with a − 0.55 ng/mL difference in BDNF concentration (95% CI -0.93, −0.16) (Figure 12A; Supplementary Table S3).

**Figure 12:**
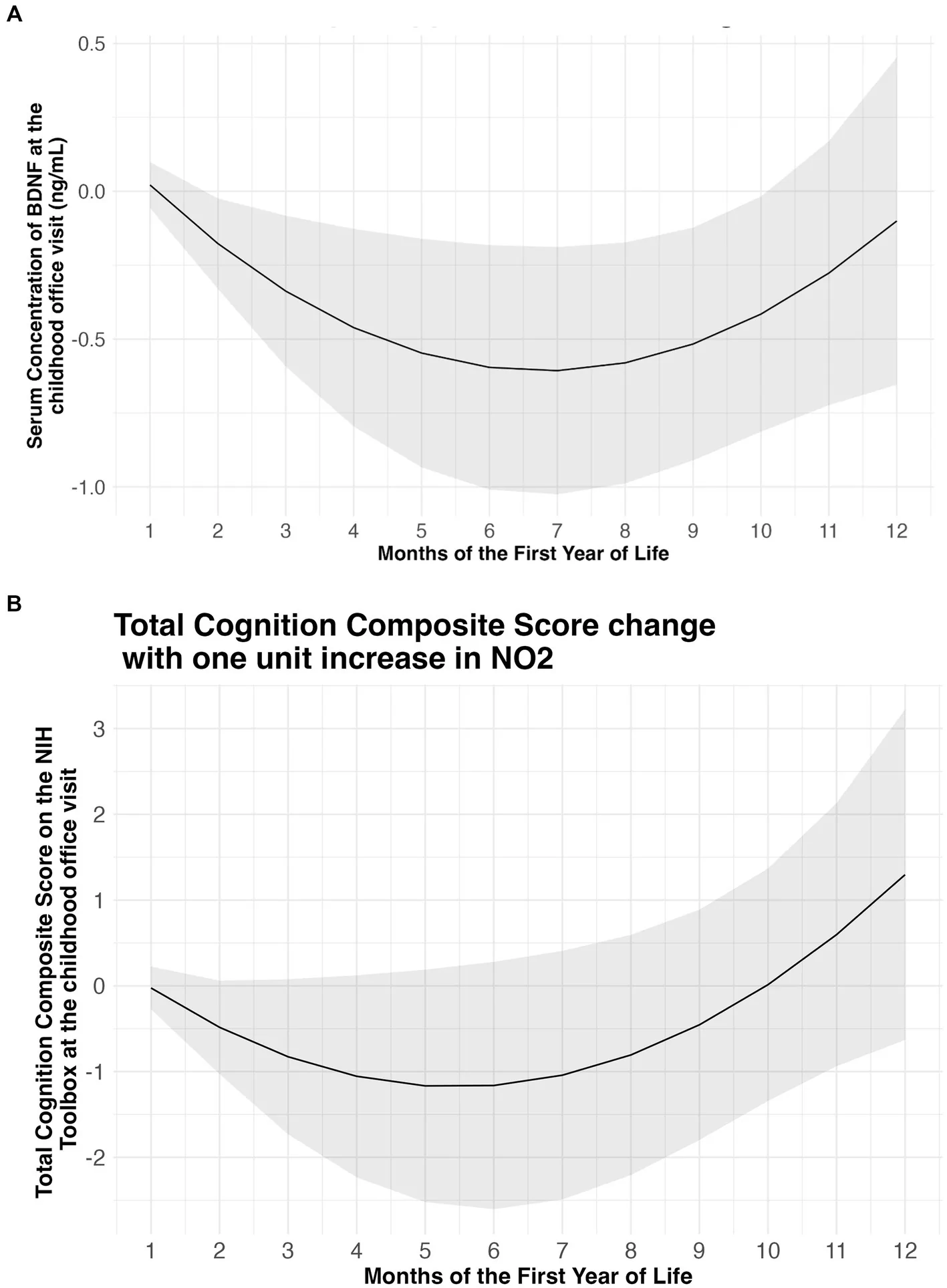
Critical windows of exposure during the first year of life and associations between: **(A)**. Brain-derived neurotrophic factor concentrations and NO_2_, **(B)**. Total cognition composite score and NO_2_.

Though an overall effect had not been seen in the linear models assessing average first year of life exposure and NIH Cognition Toolbox scores, in the DLM models the middle months of the first year of life showed a pattern of negative association with NIH Cognition Toolbox scores. The pattern of effect estimates relating NO_2_, NOx and PM_2.5_ to cognition showed a very similar bowl shape with a minimum around the middle of the first year of life to those with the BDNF concentrations. For example, a 1 ppb increase in NO_2_ during the child’s fifth month of life was associated with a −1.33 change in total cognition score (95% CI −2.64, −0.022) (Figure 12B; Supplementary Table S3).

## 4 Discussion/interpretation

The findings of this study can have an impact much larger than might be expected for a study of this size. While there were no clear relationships seen between pregnancy/early-life pollutant exposures and the detailed cognitive assessment, that is perhaps unsurprising given the small sample size (<200 subjects) and the long duration between exposure and the measured outcome. Yet, this analysis demonstrated key relationships between the California state standardized testing scores that our participants reported from their school-based testing, and more in-depth neurobehavioral assessments performed at our field office. Most notably, there was a strong relationship between 3rd grade SBAC testing scores and NIH Toolbox Cognition Battery scores. Because the NIH Toolbox is intended as an age-agnostic assessment tool, utilizing the 3^rd^ grade testing scores in this study (rather than restricting to participants with recent school standardized testing) allowed for the largest possible sample size and would be expected to relate well to Toolbox scores, even though the NIH Cognition Toolbox was completed at a wider variety of ages. For those that had recent testing, there were also moderate relationships between *recent* SBAC scores and the NIH Toolbox scores, as well as recent SBAC scores and parent-reported social or behavioral problems. Finally, there was also a moderate correlation between serum BDNF and recent SBAC scores. These relationships suggest that SBAC scores—a much more easily ascertained measure, and one that is available for nearly all California students—could potentially be used as a proxy outcome, if these findings are replicated in additional cohorts. While imperfect, these results suggest that SBAC scores are highly related to other cognitive, social and behavioral outcomes, and suggest that future work should investigate whether interventions aimed at improvements in SBAC could improve these other measures as well.

A recent study from our group demonstrated that when assessed using all repeated measures available from this cohort, an individual participant’s SBAC scores were significantly and inversely associated with exposure to PM_2.5_ in the year prior to testing, with a stronger effect for math than for English/language arts (64). A 1 μg/m3 ^3^ increase in prior year PM_2.5_, considering their exposures at home and school, was associated with 7 to 8 point lower raw SBAC scores- a sizeable amount given that the difference between the grade level means is roughly 20 points in middle school and 35 points in elementary school. In a different analysis (using statewide data from thousands of California schools and millions of students), fewer students meeting state standards for SBAC scores (per school) were associated with pollutant concentrations at each school, particularly with those two years prior (84). Taken together, these studies suggest some important possibilities. This seems to be an instance where ecologic data are a good reflection of associations at the individual level; air pollutant exposures were related both to lower individual student SBAC scores and fewer students at a given school meeting the standards. Plus, from this analysis the individual SBAC scores are strongly related to more in-depth neurocognitive testing. This combination of findings suggests that school-level standardized testing results might be a potentially useful metric for evaluating outcomes of air pollutant policy changes for California children. Changes in SBAC test scores could be both a target of interventions, and because the data are widely available, they could also potentially be used for future impact assessments. Because of the large sample size in state standardized test result datasets, small effects are more likely to be captured, and even small changes in neurobehavioral functioning may be important when considering the population distribution.

Even though we did not find a relationship between average pregnancy or early-life exposures and results from NIH toolbox testing, there were some relationships with child behavior checklist items. While we are cautious to not overinterpret individual confidence intervals that avoid the null—given the large number of parameters studied—there are two parent-reported behavior concerns that seem to show general patterns of increase in association with pollutant exposures in pregnancy. First trimester exposure to traffic-related air pollutants (nitrogen oxides, particulates, PAH) may be a contributor to rule-breaking and aggressive behavior in childhood and adolescence.

Associations between first year of life pollutant exposures and SBAC scores were generally null, and not noteworthy, even though prior analyses had found relationships with more recent pollutant exposure. There was a pattern of associations found with pregnancy exposures and SBAC test scores. However, subgroup sensitivity analyses demonstrated that age at kindergarten entry may be an important confounder of that relationship. Pollutant exposure in Fresno has a strong seasonal pattern, with traffic-related pollutants being the highest between November and February, and ozone being the highest in June through September. Thus, because pregnancy covers only a portion of the year, the pregnancy concentrations are strongly related to when the child was born, and thus, when they started school. It is well established that, in general, children within the same grade who are older than their peers have higher educational achievement (85). Thus, amongst the full cohort, the high traffic related air pollutant exposures and lower ozone exposures that occur during the winter may be serving as a proxy for being an older student, manifesting in what looks like higher test scores associated with higher pollutant exposures.

When comparing the younger students to the older students, not only does the traffic related air pollutant-SBAC score relationship invert, but so does the association with ozone. Because of (1) inverted associations between children born ~ half a year apart, and (2) the ozone relationship consistently opposing the relationship with the other pollutants, this pattern of results is consistent with the pollutant exposures serving as a marker for a seasonal pattern. Between the age strata, older children have an academic advantage and are also more likely to have high traffic-related pollutant exposure and low ozone exposure. *Within* each age stratum, the older children still have an academic advantage. But the two different strata have different pollutant exposure patterns consistent with being older *within* the stratum, and this manifests as opposite associations between SBAC scores and pollutants between the two strata, implying that this initial, unstratified finding is confounded. Thus, in total, we did not find noteworthy relationships between SBAC scores in third grade and pregnancy or first year of life air pollution exposure.

Lower BDNF serum concentrations in childhood and adolescence were associated with exposure to all seven pollutants during the first year of life. Distributed lag analysis allowed for assessment of the periods of exposure with the strongest estimated associations, demonstrating the strongest effect size around age 6 to 7 months. This pattern could result from a combination of factors including that the first 6–8 months are a period of the most rapid synapse formation in the brain (86) and that by age 6 months, more babies may be spending time in less controlled environments and being exposed to more air pollution. While this is not sufficiently supported by this study, the similar pattern of traffic-related air pollution associations with BDNF and NIH Toolbox scores raises the provocative possibility that changes in BDNF could be mediating later cognitive effects, and should be further evaluated.

Recent studies on the demonstrated effects of PM_2.5_ on neurodevelopment using laboratory and animal data provide a strong biologic plausibility for a link between air pollutant exposure and neurodevelopment in children (87). For example, PM is directly toxic to human neurons (88), can change neuron gene expression (89, 90), and may also have a role in central nervous system myelination (91). These direct effects are problematic given that particulate matter (especially the ultrafine particulate fraction in EC, those less than 0.1 microns in diameter) can enter the systemic circulation, can cross the blood–brain barrier, and have been found in the brain tissue (87, 92). In addition to the direct effects of pollutants, the induction of reactive oxygen species and oxidative stress leading to inflammation (93) is known to contribute to the pathogenesis of respiratory disease, cardiovascular disease, and cancers. Oxidative stress has been implicated in neurodegenerative disorders (94, 95) and is also thought to be a potential key contributor to difficulties in learning and cognition (96, 97). Rodents have demonstrated increased neuroinflammation following exposure to fine particles, including prenatal exposure (98–100). In people, exposure to prenatal diesel exhaust, which has a large PM component (87), induced long-term changes in the microglia, the resident immune cells in the brain, into adulthood (98), mirroring the animal studies. Nitrogen oxides and ozone directly induce oxidative stress in their original state or can decompose to free radicals, while pollutants like PM and PAH create oxidative stress through tissue inflammation and/or damage in biological systems. Free radicals contribute to damage of several cellular and subcellular structures, including lipid membranes and DNA (96). Youth with asthma are particularly susceptible; air pollution can trigger symptoms, driven by airway inflammation (101) that also affects the systemic circulation.

Yet, air pollution is only one of many factors that likely influence children’s cognition and neurodevelopment (102). Because, the structure and functional connectivity of the brain change substantially in fetal and postnatal life, many factors during this period may have long-lasting influences on neurodevelopment, including prenatal parental stress, substance use and infections (103). Nutrition is a particularly key component, and ensuring both adequate nutrition and intake of specific micronutrients is key protective factor for brain development. For example, the fortification of salt greatly decreased the prevalence of iodine deficiency (104), particularly in low socioeconomic status (SES) populations, who are likely to also have higher air pollutant exposures. This is especially key since air pollution and SES have been shown to have interactive effect on children’s health, including interactive associations that have been found for prenatal exposures in the same geographic area as our cohort (105). Future replications of these findings could include interactions with other factors, in order to best tailor interventions.

A major strength of this analysis is the inclusion of a wide array of different neurobehavioral outcomes in a population of youths who are highly exposed to air pollution. The strong associations demonstrated between the different outcomes are important for considering the potential use of standardized testing as a proxy outcome that can be targeted for air pollution interventions. Given that this cohort is from highly mobile population, persistence in our longitudinal cohort study may, itself, create a selection bias where those who remain share characteristics that are different from those who left the study. Though those who left the study do not differ from those that remained based on air pollution exposure (64), the differences in covariate distribution may limit the generalizability of these results for the most mobile, and lowest socioeconomic status families. As always, use of home-based pollutant estimates has inherent exposure misclassification based on spatial distributions, though it avoids the misclassification resulting from using personal monitoring over a short-time period to estimate exposure for a longer one. Because this population is of relatively similar SES and from a small geographic area, it is unlikely that this misclassification is differential by outcome status, since it is years prior to the measured outcomes. Yet, because there is a long lag between the exposure period and outcome assessment, the biomarkers, surveys and cognitive testing are likely all additionally influenced by short term factors such as recent nutrition, sleep, inflammation or infections (106). Though this study is of a smaller sample size, which may have limited the ability to find associations between pregnancy and early-life air pollutant exposures and neurobehavioral outcomes, this small sample comes from some of the most impacted youth in California, and some associations were apparent.

Air pollution effects on childhood social–emotional, behavioral and cognitive development have a strong basis in pathophysiology and have been identified in several studies. The key contributions of this study are that California SBAC testing was correlated with intensive cognitive testing in youths, a biomarker of neurogenesis, and parental reports of emotional and behavioral difficulties; and exposure to air pollutants in pregnancy and during the first year of life were associated with neurocognitive and behavioral effects that extend into late childhood and adolescence.

## Data Availability

Deidentified datasets generated for this study are available upon request from the authors.
